# TRIM28 Selective Nanobody Reduces Glioblastoma Stem Cell Invasion

**DOI:** 10.3390/molecules26175141

**Published:** 2021-08-25

**Authors:** Andrej Porčnik, Metka Novak, Barbara Breznik, Bernarda Majc, Barbara Hrastar, Neja Šamec, Alja Zottel, Ivana Jovčevska, Miloš Vittori, Ana Rotter, Radovan Komel, Tamara Lah Turnšek

**Affiliations:** 1 Department of Neurosurgery, University Medical Centre Ljubljana, 1000 Ljubljana, Slovenia; andrej.porcnik@kclj.si; 2 Department of Genetic Toxicology and Cancer Biology, National Institute of Biology, 1000 Ljubljana, Slovenia; metka.novak@nib.si (M.N.); barbara.breznik@nib.si (B.B.); bernarda.majc@nib.si (B.M.); barbara.hrastar@gmail.com (B.H.); ana.rotter@nib.si (A.R.); 3 Jožef Stefan International Postgraduate School, 1000 Ljubljana, Slovenia; 4 Medical Centre for Molecular Biology, Institute for Biochemistry and Molecular Genetics, Faculty of Medicine, University of Ljubljana, 1000 Ljubljana, Slovenia; neja.samec@mf.uni-lj.si (N.Š.); alja.zottel@mf.uni-lj.si (A.Z.); ivana.jovcevska@mf.uni-lj.si (I.J.); 5 Department of Biology, Biotechnical Faculty, University of Ljubljana, 1000 Ljubljana, Slovenia; milos.vittori@bf.uni-lj.si; 6 Faculty of Chemistry and Chemical Technology, University of Ljubljana, 1000 Ljubljana, Slovenia

**Keywords:** glioblastoma, nanobody, glioblastoma stem cells, cell invasion, transcription factor, TRIM28

## Abstract

Glioblastoma (GB), is the most common and aggressive malignant primary brain tumour in adults. Intra- and inter-tumour heterogeneity, infiltrative GB cell invasion and presence of therapy-resistant GB stem cells (GSCs) represent major obstacles to favourable prognosis and poor therapy response. Identifying the biomarkers of the most aggressive tumour cells and their more efficient targeting strategies are; therefore, crucial. Recently, transcription factor TRIM28 has been identified as a GB biomarker and, in this study, we have shown high expression of TRIM28 in GB and in low grade gliomas as well as higher expression in GSCs vs. differentiated GB cells, although in both cases not significant. We demonstrated significant in vitro inhibition of GB cells and GSCs invasiveness and spread in zebrafish brains in vivo by anti-TRIM28 selective nanobody NB237. TRIM28 was also enriched in GB (tumour) core and associated with the expression of stem cell genes, but was not prognostic for overall survival. However, based on the above results, we conclude that TRIM28 nanobody NB237 offers a new opportunity as a GB therapeutic tool.

## 1. Introduction

Glioma WHO grade IV, termed glioblastoma (GB), is the most common, and most aggressive malignant primary brain tumour in adults. The standard of care treatment for newly diagnosed GB relies on maximal surgical resection, followed by irradiation and concomitant chemotherapy with the alkylating agent temozolomide (TMZ) [1]. Most recently, the sixth version of the international standard for the WHO Classification of Tumors of the Central Nervous System (CNS) [2] and European Association of Neuro-Oncology (EANO) suggestions [3], stated that the classification of GB is not only based on histology but also on the expression of several molecular markers and is now defined as a diffuse astrocytic glioma with no mutations in *IDH* genes nor *histone H3* genes mutations. Grade IV astrocytoma is genetically distinct from a much more common IDH-wild type GB, although its histological appearance is similar. Based on clinical progression and survival rate, gliomas are also classified into high grade gliomas (HGG) (i.e., WHO grade III and IV gliomas), and low grade gliomas (LGG) (i.e., the WHO grades I–II gliomas) [4].

Despite the aggressive GB treatment protocols, the overall median survival is still less than two years, as most patients eventually develop resistance to therapy, resulting in recurrent tumours. The main reasons of therapeutic failure are GB heterogeneity, invasion and the presence of a small population of therapy-resistant glioblastoma stem cells (GSCs) [5,6,7]. Recent discoveries revealed that according to their molecular profiling, three major GB subtypes exist, proneural (PN), classical (CL) and mesenchymal (MES) [8,9], each associated with a specific driver of genetic alterations. The major ones include activation of mutated platelet-derived growth factor receptor alpha (PDGFRA), OLIG2, TCF3 and IDH mut in PN, alterations in epidermal growth factor receptor (EGFR) in CL subtype and neurofibromin 1 deletions, along with MET, CD44, CHI3LI/YLK40 in MES [9]. The three GB subtypes have also different prognostic and therapeutic (i.e., predictive implications), among which the MES GB subtype is the most aggressive and reportedly associated with the poorest prognosis [10]. Furthermore, Patel et al. [7] established that GB subtype-specific markers may be variably expressed across individual cells within one tumour, comprising a mixed (MIX) GB subtype, representing the highest level of intra-tumour heterogeneity and associated with the worst survival of GB patients.

GSCs are a small subpopulation of cells within tumours with abilities to self-renew, give rise to differentiated progenies, as well as initiate and sustain tumour growth. GSCs are mostly present in a dormant/quiescent state, but are mobilized to proliferate due to exogenous stimuli by stromal cells and other microenvironmental clues, including cytokines/chemokines, growth factors and proteases. Furthermore, innate cues may induce GSCs motility, enabling their migration and invasion into brain parenchyma [11,12,13]. The key hallmark of GB cells is; thus, diffuse, infiltrative invasion on a single cell level, representing another major obstacle for curative therapy [11,12]. Invasive GB cells are the most difficult “moving” targets to address. Promising anti-invasive tools are nanobodies that block invasion-related genes. Nanobodies are variable domains of the functional heavy-chain antibodies, size 12–14 kDa, naturally occurring in the serum of Camelidae species [14]. Soluble nanobodies have distinct characteristics, such as solubility, nanometre dimensions and very strong affinity for their antigens [15]. Moreover, nanobodies and the VH domain of family III human immunoglobulins have 80% sequence homology [16], meaning that nanobodies present with low immunogenic risk and are; therefore, suitable for therapeutic application in humans, such as in for solid cancers therapy [17,18]. In addition, nanobodies are considered suitable for treating GB and other central nervous system (CNS) pathologies, since they have been reported to be able to pass the blood–brain barrier (BBB) [19,20,21,22,23]. In this regard; however, only two studies have been reported so far. Roovers et al. used anti-EGFR nanobodies in an in vivo murine xenograft model and showed promising results for further investigations [24]. Furthermore, US28-targeting nanobodies that significantly impaired human cytomegalovirus (HCMV)/US28-mediated glioblastoma growth in vitro and in vivo have been developed [25]. One of the possibilities to circumvent BBB is direct nanobody application to the tumour during surgery. In this case, larger amounts of a nanobody can be applied without additional potential off-target effects.

TRIM28 together with TRIM24 and TRIM33 belongs to the sixth subfamily that is involved in the control of gene expression by regulation of transcriptional activity of numerous sequence-specific transcription factors. All three proteins are involved in the processes of cell growth, development and differentiation [26], whereas their alterations have impact on transcriptional regulation, cell proliferation and apoptosis [26]. TRIM proteins are known to play different roles in tumorigenesis [26]. TRIM28 has been reported to enhance autophagy and promote GB cell proliferation in vitro [27]. Although current findings implicate an oncogenic activation of TRIM28 in GB, the exact molecular mechanism of its function is yet to be determined. In our previous work, we have identified and validated the nanobody referred to as NB237 [28,29] that targeted TRIM28. This is also known as transcriptional intermediary factor 1-β (TIF1-β) or KRAB interacting protein 1 (KAP1) [30,31,32]. 

The first aim of our study was to compare the gene expression of *TRIM28* in a cohort of 95 GB and 19 LGG tumour samples and non-cancerous brain samples, as well as in primary patient-isolated differentiated GB cells vs. their GSC counterparts. Secondly, we also addressed possible association of *TRIM28* expression with the GB subtypes. Thirdly, we evaluated *TRIM28* expression as a potential prognostic biomarker. Lastly, we aimed to test the recent hypothesis by Minata et al. [33], reporting the difference among GB cells in invading edge (rim) of the tumour and the cells in GB core that was related to their genotype, the rim-bearing cells resembling the recurrence-initiating and more invasive cells. These GB cells may express higher *TRIM28* levels, as indeed confirmed in the present study. TRIM28 thus seems to represent a target for GB invasion, being enriched in the tumour core with MES subtype–related and stem cell genes. Finally, we validated this hypothesis, testing the nanobody NB237 targeting TRIM28 protein for the inhibition of differentiated GB cell and GSC invasion in the in vitro as well as in in vivo system using zebrafish. Zebrafish embryos have already developed brain structures, which mimic the cell microenvironment in humans. The model has several advantages over rodents representing fast, high-throughput and low-cost animal experimental system. We have already proven that the xenotransplantation of GB cells into the brain of zebrafish embryo is a great tool for studying GB growth and invasion in a complex brain microenvironment in vivo [34,35,36,37].

## 2. Results

### 2.1. TRIM28 Gene Is Enriched in GSCs and CL GB Tissue Subtype

**Figure 1:**
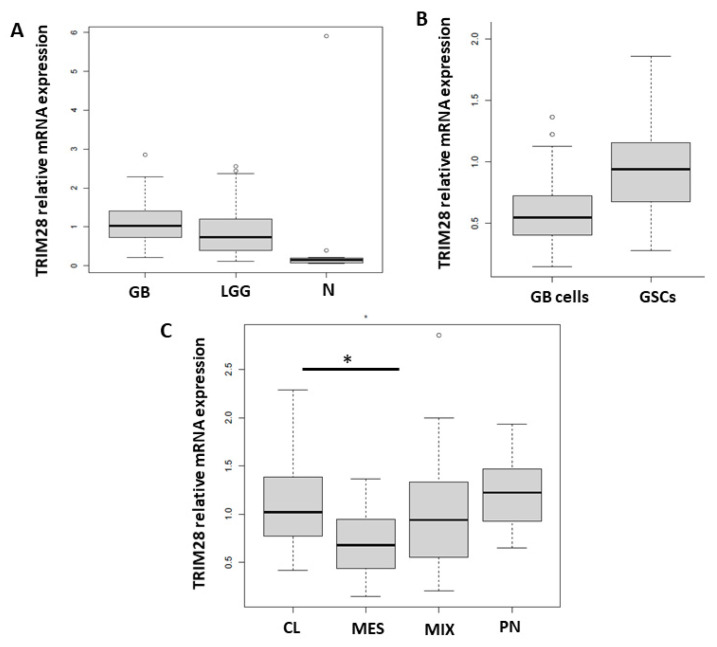
TRIM28 gene expression in glioma tissues, GB cells and GB tissue subtypes. TRIM28 mRNA expression levels were determined by RT-qPCR in (**A**) GB (*n* = 95), LGG (glioma I–II, *n* = 19) and non-cancerous brain tissues (N, *n* = 16). (**B**) Primary differentiated GB cells (GB cells, *n* = 18) and GSCs (*n* = 6), and (**C**) CL (*n* = 24), PN (*n* = 8), MES (*n* = 4) and MIX (*n* = 59) GB tissue subtypes. mRNA values were normalized to housekeeping genes HPRT1 and GAPDH and analysed with quantGenius software [41]. Statistical analysis was performed in R, using analysis of variance to determine the homogeneity of variance between glioma tissues, primary and stem cells, and subtypes. Tukey’s post hoc test was then used to for multiple comparison between groups with significance levels (* *p* < 0.05). Different *Y*-axis scales are presented.

We have shown before, on a protein level, that TRIM28 is expressed in glioblastoma tissues and glioblastoma stem cells [28]. Here, the expression of the *TRIM28* gene was evaluated as a possible biomarker and prognostic marker for GB. We determined the mRNA expression levels of *TRIM28* in the tissues of a normal non-cancerous brain (N) (*n* = 16), GB (*n* = 95) and LGG (glioma I–II, *n* = 19). Moreover, 18 primary differentiated GB cells and six GB stem cella (GSCs) were established from GB tissues. *TRIM28* mRNA levels were higher in malignant specimens compared to non-cancerous brain tissues (Figure 1A). GSCs expressed higher levels of *TRIM28*, than the primary GB cells (Figure 1B), although this difference did not reach significance, presumably due to uneven and low numbers of GSCs vs. differentiated GB cells. Furthermore, we analysed *TRIM28* levels in the four GB subtypes, MES, PN, CL and mixed (MIX). This classification was based on the expression values of 12 subtype-specific genes according to Behnan et al. [38], to which we added three more genes based on in house transcriptomic analyses [39] (Tables S1 and S2). PN subtype was classified by the expression levels of *OLIG2*, *P2RX7*, *STMN4*, *SOX10*, *NOTCH* and *ERBB3* genes. CL subtype was classified by the expression levels of *NF-KB, ACSBG1, S100A4* and *KCNF1* and MES subtype by the expression levels of, *DAB2*, *TGFB1*, *THBS1*, *COL1A2* and *COL1A1*, as described in Materials and Methods. With respect to the level of *TRIM28* mRNA, CL subtype exhibited the highest, whereas MES subtype expressed the lowest level of its expressions (Figure 1C). To clarify these findings, we made additional analysis with the larger cohort of 156 GB samples, deposited in TCGA GlioVis database [40] (http://gliovis.bioinfo.cnio.es/, accessed on 22 August 2021, Supplementary data, Figure S1 in Supplementary Materials). We confirmed TRIM28 was significantly expressed (*p* < 0.01) in GB samples and significantly in CL GB subtype (*p* < 0.01), compared to MES GB subtype (Figure S1).

### 2.2. TRIM28 Is Enriched in GB Core of MES Subtype and Stem Cell Genes’ Expression

**Figure 2:**
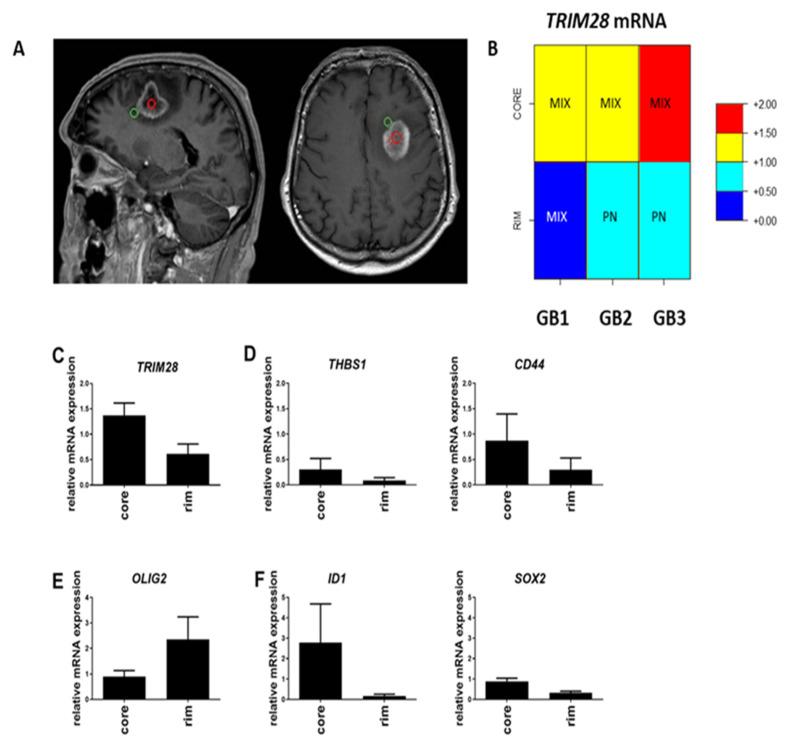
Gene expression analysis of the GB core versus rim areas. (**A**) T1-weighted MRI scans of a representative patient GB3, with multi-region surgical sampling. Regions include central enhancing core in red circle and rim area as the invasive edge in green circle, defined by the 5-aminolevulinicacid (5ALA) fluorescence positive area beyond the enhancement, according to the image guidance navigation system. (**B**) GB subtype analysis of core and rim areas in three GB samples (GB1, GB2 and GB3). High or low expression of *TRIM28* within those areas is scaled in colours, representing relative expression (normalisation) of mRNA levels of *TRIM28* gene vs. the mean of two housekeeping genes, *HPRT1* and *GAPDH.* The red colour in Figure 2B visualizes relatively higher levels *of TRIM28* mRNA expression (>1), while the blue colour indicates lower *TRIM28* mRNA values (<1), and these are clearly separated in the rim vs. core tissues, shown separately in each GB1, GB2 and GB3 samples. (**C**) Expression of *TRIM28*, (**D**) MES subtype genes (*THBS1, CD44*), (**E**) PN subtype gene *OLIG2*, and (**F**) GSC genes (*ID1*, and *SOX2*), were analysed in GB tissue core and rim areas, by RT-qPCR. mRNA values were normalized to housekeeping genes *HPRT1* and *GAPDH* and analysed with quantGenius software [42]. Presented results are the mean ± S.D. of three GB samples.

Deeper insight into intra-tumour heterogeneity and subtype differences in GB cells located in the core or at the rim (i.e., the diffuse invading edge of the tumour) (Figure 2A) are not well explored. It is very important to explore the genotype of GB cells that are located at the edge of the tumour that invade, leading into the recurrence of the disease. We analysed whether the *TRIM28* mRNA expression levels at the rim and in the core of the tumour tissues differ in 3 GB samples (GB1, GB2, GB3). *TRIM28* mRNA expression levels were higher in the core of the GB samples (Figure 2B). The core is enriched in the MES subtype, expressing higher *THBS1* and *CD44* marker genes (Figure 2C). The PN subtype marker *OLIG2* gene was highly expressed at the rim of GB (Figure 2D). Beside enriched MES genes in the core, GB stemness-related genes *ID1* and *SOX2* were also highly expressed in the core (Figure 2E,F). Overall, the core of GB tissue was enriched in MIX subtype and rim of GB tissue is more PN subtype (Figure 2A).

### 2.3. TRIM28 Was Not a Prognostic Marker of GB Overall Survival

**Figure 3:**
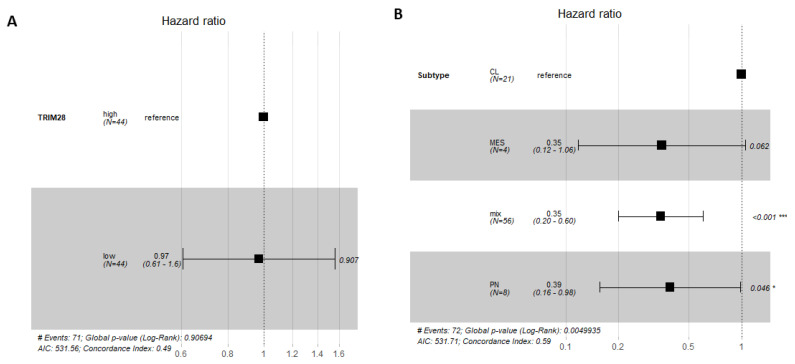
*TRIM28* is not a prognostic marker of GB patient survival. Forest plot for Cox proportional hazards model showing the association between *TRIM28* expression levels in GB and GB subtypes and survival, with confidence interval of 95% CI. (**A**) *TRIM28* high or low expression, within GB group (*n* = 88). (**B**) *TRIM28* high or low expression, within CL, PN, MES and MIX GB subtypes, has no effect on prognosis of GB patient survival. CL GB subtype is set as a reference. * *p* < 0.05, *** *p* < 0.001.

A Forest plot array with a confidence interval of 95% CI, represented by the black squares and horizontal lines, which reflect the weight of each sample for multiple analysis, was used to analyse whether high or low *TRIM28* expression in GB patient samples has an effect on prognosis of GB patient survival (Figure 3). Hazard ratio (HR), the estimated risk factor, was calculated for *TRIM28* expression above or below median value, within GB (*n* = 88) group. Neither *TRIM28* high nor low expression (HR = 0.97) is a prognostic marker in GB group (Figure 3A). *TRIM28* had also no significant prognostic potential at top 25% high and bottom 25% *TRIM28* expression levels (Figure S3). TRIM28 high or low expression, within CL, PN, MES and MIX GB subtypes, has no effect on prognosis of GB patient survival. MIX GB subtype has significant value in having better survival prognosis among GB subtypes, regardless of TRIM28 expression (Figure 3B).

### 2.4. TRIM28 Nanobody Decreases In Vitro Invasion of GSCs and of GB Cells

**Figure 4:**
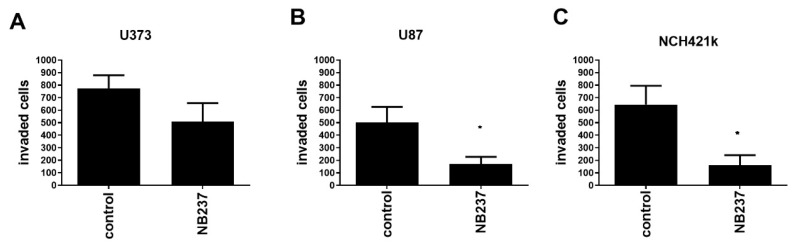
Effect of TRIM28 nanobody NB237 on invasion of GB cells. (**A**,**B**) GB cells, U373 and U87, at 10,000 cells/insert; and (**C**) GSCs NCH421k at 80,000 cells/insert, were seeded in the upper compartment in serum-free medium alone or in combination with TRIM28 nanobody (final concentration 100 µg/mL). The cells that invaded the Matrigel after 48 h were stained with 0.1% crystal violet and counted using inverted microscope. Each value represents mean ± S.D. (Three biological replicates). Statistical analyses was performed using GraphPad Prism software, using *t*-test, * *p* < 0.05, vs. control group.

We demonstrated the relevance of TRIM28 protein expression for GB cells invasion and confirmed the functional significance of the TRIM28 nanobody. Transwell invasion assays using Matrigel were used to measure the invasion of GB cells, U373 and U87, and NCH421k GSCs, prior and post treatment with TRIM28 nanobody (NB237) added in the upper chamber (Figure 4). TRIM28 nanobody in the highest not cytotoxic concentration in vitro was used [29]. The invasion of U373 cells was not significantly inhibited (Figure 4A), whereas invasion of U87 cells was significantly and highly (up to 65%, Figure 4B) inhibited, similar to NCH421k, where TRIM28 nanobody treatment reduced invasion by 70% (Figure 4C). 

### 2.5. The TRIM28 Nanobody Inhibited Invasion of GB Cells and GSCs in an In Vivo Assay in Zebrafish Embryos

**Figure 5:**
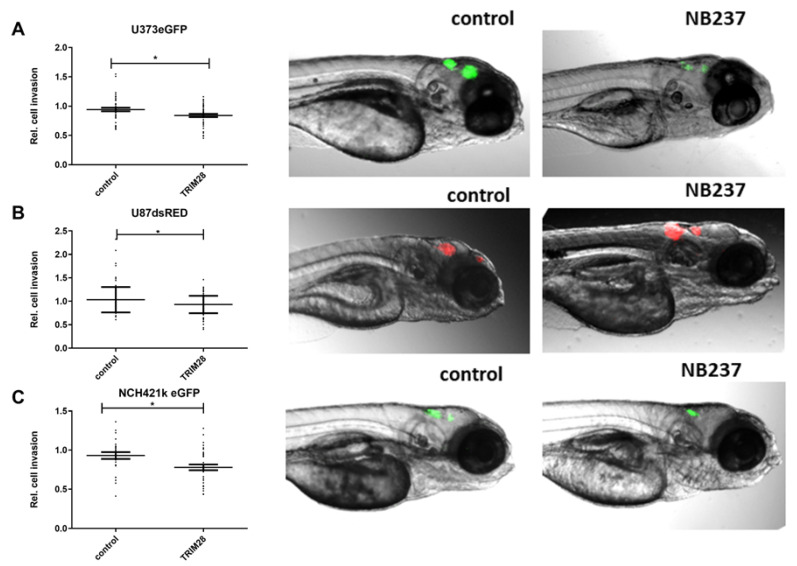
In vivo imaging of GB cell and GSC invasion in the brain of zebrafish embryos. Embryos 72 h after the xenotransplantation of (**A**) U373eGFP, (**B**) U87DsRed and (**C**) NCH421k eGFPcells in the brain (visible as red and green fluorescence) without (control) or with the TRIM28 nanobody NB237. Data are shown as means ± S.D., dots on graphs (**A**–**C**) represent each embryo. GB xenograft diameter at 72 h was normalized to GB xenograft diameter at 24 h after xenotransplantation to determine the relative cell invasion. Number of embryos in control and with NB237 in the experiment with U373 *n* = 37–50, U87 *n* = 94–108, NCH421k *n* = 13–38. Statistical analyses were performed using GraphPad Prism software, using Mann–Whitney test, * *p* < 0.05, vs. control group.

We injected fluorescent GB cells and GSCs alone or together with TRIM28 nanobody NB237 into the brains of zebrafish embryos, as described in the Methods. GB cells, U373 and U87, and NCH421k GSCs were successfully injected in zebrafish embryo brains and formed compact tumours in the midbrain of the brain after 24 h (Figure 5). Relative cell invasion, determined as a change in GB xenograft length between 72 h and 24 h after xenotransplantation was calculated as described in Methods. Relative invasion of U373 cells in zebrafish embryo brain with TRIM28 nanobody significantly decreased for 15% (Figure 5A) and for 13% in U87 cells after addition of TRIM28 nanobody (Figure 5B). NCH421k cells with TRIM28 nanobody showed significant 25% decrease in invasion as compared to GSCs alone (Figure 5C), which is in line with the in vitro observations (Figure 4C). In contrast, relative tumour growth in zebrafish embryo brain, determined as a change in mean fluorescence intensity of GBM xenografts, after addition of TRIM28 nanobody was not altered (Figure S4), implicating that a decrease in GB xenograft length is indeed a consequence of impaired cell invasion.

## 3. Discussion

TRIM proteins can either promote or suppress oncogenes and tumour progression by affecting various cellular physiological processes such as DNA repair, cell proliferation, autophagy and apoptosis [14,27,43,44]. Transcription factor TRIM28 is a part of tripartite motif (TRIM) containing a protein superfamily, which contains approximately 70 members [30,31,32]. Detected in cell nuclei but not in cytoplasm, TRIM28 serves as an important factor in the posttranslational mechanism to regulate various cell functions, such as transcriptional regulation of p53 suppression by promoting its ubiquitination and degradation [44,45]. In addition, TRIM28 has an important role in the oncogenesis in different cancer types [46,47]. TRIM28 expression is elevated in a variety of tumours, including breast cancer, gastric cancer and lung cancer [46,47,48,49]. In a clinical study of patients with early-stage lung tumour, TRIM28 expression significantly correlated with overall survival [48]. In GB, it has been shown by others [43] that TRIM28 gene is overexpressed compared to LGG and to non-cancerous tissues. However, in contrast to Qi et al. [43], who in a large cohort of 483 glioma patients showed that TRIM28 overexpression is positively associated with poor patients’ prognosis, we could not find TRIM28 mRNA expression to be prognostic in a cohort of 88 samples of the highest glioma grade in GB patients. To validate this finding in the larger cohort of 155 GB samples, we tested the deposited GB data in GlioVis database [40] (http://gliovis.bioinfo.cnio.es/, accessed on 22 August 2021, Supplementary data, Figure S2). We found that TRIM28 was also not a prognostic marker for overall survival in 155 GB patients. 

Further, we studied the intra-tumoural heterogeneity in GB samples, where the characteristically diffuse rim of the tumour was separated from its core by neurosurgical operative imaging. Molecular analysis of the three patient-derived GB core and rim tissue samples revealed that the tumour regions differ in their genotype. We found that the core is enriched in MES subtype-associated genes along with higher levels of the GSC stemness markers, whereas the PN markers were more expressed in the rim, that seem contradictory to the hypothesis of invasive MES GBM subtypes cells localised at the invasive tumour edge. However, this could be explained by the finding of Bastola et al. [50] on spatially distinct gene expression in glioblastoma edge (cells spheroids, mouse xenografts and human tissues), showing a great degree of PN-GB signatures at the edge/rim, whereas the core-associated cells were a mixture of cells with all three GB subtypes, including MES-GB signature. These authors stated that there is no question that the edge-located tumour cells subsequently develop lethal recurrence, but also show a higher capacity for infiltrative growth, whereas core cells demonstrate some lesions with strong therapy resistance. Importantly, this study identified core cells that disseminate some of their malignant properties, to less aggressive edge/rim GBM cells, by passing a number of extracellular signals to support their ecosystem by paracrine cross-talk among GB cells only, independently of their microenvironment. In conclusion, their study demonstrated the presence of homotypic inter-cellular signals that affect tumour-initiating cells at the edge and promoted growth and radio-resistance of the edge/rim counterparts, leading to the appearance of stable, cell autonomous differences between core- and edge/rim-located GBM cells. This phenomenon may have more relevant clinical significance by characterising spatial cells genotyping vs. discriminating among overall GB subtyping, which so far has not shown great clinical or therapeutic relevance. 

An altered cancer stem cell marker profile in GB core and rim has already been suggested by Smith et al. [51]. We also found GSC stemness-related genes, SOX2 and ID1, and MES subtype-related genes, THBS1 and CD44, enriched in the core of the tumours, together with higher TRIM28 expression. This suggests association of TRIM28 expression with GSCs, homing to the core of the tumour and corroborated with the previous report on altered cancer stem cell marker profile in GB core vs. rim [51]. Here, we are also confirming our previous work [28,52] showing higher TRIM28 expression in GSCs as in GB cells. The role of TRIM28 in GSCs has been elucidated recently by Zhang C. et al. [53], when using experimental knockdown of TRIM28 that reduced GSC self-renewal and promoted glial differentiation. Further, these authors found that TRIM28 activates STAT3 by suppressing its inhibitor PIAS3, which most likely triggers E3-mediated ubiquitination and proteasomal degradation. Reciprocally, STAT3 activation upregulates TRIM28 and normalizing TRIM28 expression. These data demonstrate that bidirectional TRIM28-STAT3 signalling regulates GSCs stemness. The rim regions, enriched with invading cells, were mostly expressing PN and MIX GB genotypes, but support the notion that TRIM28-eriched MES GB cells may have been detached from the tumour, representing the more aggressive MES-GSCs that seed novel GB islets in the brain. These data suggest a phenotypic plasticity of the GSCs in rim vs. core of gliomas [46], although a larger number of tumours are needed to support such a conclusion on the dynamics (i.e., spatial and temporal distribution of GSCs in GB tumours).

Invasiveness of GB cells is responsible for tumour local and even metastatic dissemination and relapse [11], enabling them to spread and invade to distant sides of the brain, even to the other hemisphere [54], and possibly metastasize out of the brain [13]. TRIM28 has already been suggested to be involved in invasion [48] and formation of brain metastases in early stage of non-small cell lung cancer (NSCLC) [55]. Herein we proved that TRIM28 is an active driver of GB cells and, even more so, GSCs invasiveness by selectively blocking TRIM28 using a specific camelid nanobody developed in our laboratory [28,29]. Various nanobodies have already been used in a number of clinical trials to treat different diseases [56] and in 2018 the first nanobody was adopted to treat patients [57]. 

The complex brain microenvironment can only be tested in animals. We have used zebrafish (*Danio rerio*) embryos as an alternative to vertebrate models. This model is becoming frequently used in cancer research [14,36,37,58], mostly due to the fact that the transparent embryos enable high-resolution imaging of implanted cancer cells on a single level and that have not yet developed an immune system, thus the additional immunosuppression is not needed. Zebrafish embryos are also transparent with developed brain structures, which mimic the brain microenvironment in humans, enabling high-resolution imaging of implanted cancer. Because of the close homology/orthology of the human and fish brain genomes [34], we found this model appropriate for in vivo imaging of cellular processes of GB progression in real time, and have also used it as such in previous studies [36,37,59]. We observed differential invasion inhibition of TRIM28 nanobody between the U87 and U373 cells in vitro. We have shown before [29] higher relative mRNA levels of TRIM28 in the U87 cell line might also explain higher in vitro effect of the NB237 nanobody on cell invasion. Inhibition of U373 cell invasion with the TRIM28 nanobody had significant effect in the in vivo system but not in the in vitro. Similarly, we have previously demonstrated in vivo that, when directly co-culturing these cell lines with the same stromal cells, such as human bone-marrow MSCs, U87 cell invasion was inhibited, whereas U373 cell invasion was enhanced, opposite to the cells alone in zebrafish brain [35]. The effect of the anti-TRIM28 nanobody on GSC invasion was remarkably higher compared to the effect on differentiated U373 cells. Considering that we have previously found that TRIM28 expression in a normal brain is significantly lower compared to GB tissue expression as well as lower-grade gliomas [29], the therapeutic administration of the anti-TRIM28 nanobody seems reasonable, especially since it shows the greatest effect on the invasive GSCs. Nanobodies are single-domain antibodies which are significantly smaller in size (15 kDa) than conventional antibodies and have been reported to be able to cross the BBB freely [21,22,23,60,61]. Regarding intracellular targeting, quite a few studies have already focused on a strategy for the non-endocytotic intracellular delivery of molecules by decorating them with short amino acid sequences rich in arginine amino acids, which gives them the potential to directly cross the cell plasma membrane (reviewed in [62]). However, anti-TRIM28 NB237 with pI of 9.10 proved to be a suitable intrabody, as after application in vitro fluorescence microscopy it showed its presence within the experimental cell lines (data not shown). 

Taken together, this is the first study to show that TRIM28 is involved in the glioblastoma invasion process in vitro and in vivo. As this is a pilot study, we suggest further research on GB progression in zebrafish to perform in vivo as a time- and concentration-dependent TRIM28 nanobody application. 

## 4. Materials and Methods

### 4.1. Cell Cultures

Human glioblastoma cell lines U373 (cell line listed under catalogue number 89,081,403 has been re-named as U-251) and U87 were obtained from American Type Culture Collection (ATCC, Manassas, VA, USA) and were cultured in DMEM high glucose medium (GE Healthcare, Chicago, IL, USA), supplemented with 10% (*v*/*v*) FBS, 2 mM l-glutamine, 100 IU/mL penicillin and 100 µg streptomycin. Glioblastoma stem cell line NCH421k was obtained from CLS (Cell Lines Service GmbH, Eppelheim, Germany) and expanded as spheroid suspensions in complete Neurobasal Medium (Invitrogen, Life Technologies, Carlsbad, CA, USA) containing 2 mM l-glutamine, 1 × penicillin/streptomycin, 1 × B-27 supplement (Invitrogen, Life Technologies, Carlsbad, CA, USA), 1 U/mL heparin (Sigma-Aldrich, St. Louis, MO, USA), 20 ng/mL bFGF and EGF (both from Invitrogen, Life Technologies, Carlsbad, CA, USA). Cells were transfected with the plasmid vector pCMVDsRed-Express2 and pEGFP-N1 to stably express the red fluorescent protein DsRed and enhanced green fluorescent protein eGFP, as described previously [59,63]. All cell lines were maintained at 37 °C with 5% CO_2_ and 95% humidity. All cell cultures were tested for mycoplasma contamination using the MycoAlert Mycoplasma Detection Kit (Lonza, Basel, Switzerland).

### 4.2. Glioblastoma Tissue Samples from Patients

Glioma biopsies were obtained from patients that were operated on at the Department of Neurosurgery, University Medical Centre of Ljubljana, Slovenia. We have collected 95 HGG samples, among them 89 GB and 6 GBr—recurrent GB and 19 LGG samples. We have also obtained 16 tissue samples of non-cancerous brain tissues. The study was approved by the National Medical Ethics Committee of the Republic of Slovenia (approvals no. 0120-179 190/2018/4, 0120-190/2018/23). All patients signed written informed consent for the use of tumour samples for research, obtained by the operating neurosurgeon. The clinical parameters and tumour histological and molecular characteristics for the gene expression analysis were provided by the Department of Neurosurgery and Institute of Pathology at Medical Faculty in Ljubljana. The samples were taken during the craniotomy and resection of the tumour. Tumour tissue samples were snap-frozen in liquid nitrogen and stored in the liquid nitrogen for RNA/DNA analyses. Tumour core and invading regions were taken during the craniotomy and resection of the tumour according to Smith et al. [51] and provided separately for the comparison of the gene expression signatures. Each sample was first taken from the core region of the tumour (named core), according to the enhancement area on image guidance (MRI) navigation system. The second sample was taken from the invasive edge or margin (named rim) of the initial sample and was defined by the 5-aminolevulinicacid (5ALA) fluorescence positive area beyond the enhancement, according to the image guidance navigation system. All methods used in this research study were carried out in accordance with relevant guidelines and regulations.

### 4.3. Establishment of Primary Glioblastoma and Glioblastoma Stem Cells

Primary glioblastoma and glioblastoma stem cells were established according to previously described protocol by Novak et al. [39].

### 4.4. Gene Expression Analysis

Total RNA from glioblastoma tissues was isolated using AllPrep DNA/RNA/Protein Mini Kit (Qiagen, MD, USA) according to the manufacturer’s instruction. For each sample, 1 µg of RNA was reverse transcribed using a High-Capacity cDNA Reverse Transcription Kit (Thermo Fischer Scientific, Waltham, MA, USA). High-throughput RT-qPCR was used to measure *TRIM28* expression. RT-qPCR was performed with FAM-MGB probes with Fluidigm BioMark HD System RT-PCR (Fluidigm Corporation, San Francisco, CA, USA) using 48.48 Dynamic Arrays IFC [63], where 42 samples and 24 assays (probes) were mixed pairwise in nanolitre chambers to enable parallel analysis of 2304 reactions. Visualization and analysis of qPCR results were done using the Fluidigm RT-qPCR analysis software and quantGenius software [47]. Relative copy numbers of mRNA were normalized to housekeeping genes *HPRT1* and *GAPDH*. Assays are described in Table S2. Statistical analysis were performed with one-way ANOVA in GraphPad Prism (GraphPad Software Inc., La Jolla, CA, USA).

### 4.5. Data Analysis 

#### 4.5.1. Glioblastoma Subtyping

We assessed whether the expression profiles of 15 selected genes, in particular COL1A2, COL1A, TGFB1, THBS1, DAB2, S100A4, P2RX7, STMN4, SOX10, ERBB3, ACSBG1, KCBF1, OLIG2, NOTCH and NF-KB, from 4 sample types (GB-; recurrent GB; GB cells-differentiated GB cells; GSC-GB stem cells) are suitable markers for GB subtype clustering into mesenchymal (MES), proneural (PN) and classical (CL) subtype, and finally the subtype combination (MIX). Since the number of subtypes (clusters) was known in advance, we used k-means clustering to partition the expression profiles of the selected genes in one of the four subtypes. The clustering yielded 4 samples clustered into MES, 8 samples clustered into PN, 24 samples clustered as CL and 59 samples clustered into MIX subtypes. The analysis was done as described in Novak et al. [39], using R version 4.0.3 and its libraries factoextra [64] and cluster [65].

#### 4.5.2. Differentially Expressed Genes among Tissues and Glioblastoma Subtypes

We analysed the differences in the expression of *TRIM28* among GB samples and between previously defined subtypes (mesenchymal—MES, proneural—PN, classical—CL subtype and finally the subtype combination—MIX) in the second analysis. To minimize the effect of genes with a low expression, we first removed them from the analysis by placing the Ct values > 40 as zero. We plotted boxplots to visually assess the differences and variability of *TRIM28* gene expression and then assessed the potential difference between sample types and subtypes using the analysis of variance (to determine the homogeneity of variance), followed by Tukey post hoc tests. The analyses were conducted in R version 4.0.3.

#### 4.5.3. Cox Proportional Hazards Regression 

Cox proportional hazards regression was calculated to assess survival in GB sample cohort of different subtypes. To assess any putative effect of *TRIM28* expression we categorized it using a simple rule: if individual gene expression was higher than the median, the gene expression was »high«, else »low«. All analyses were done in R version 4.0.3.

### 4.6. Expression and Purification of TRIM28 Nanobody

Identification and characterization of the TRIM28 nanobody, named NB237, was described in [28,29,51]. NB237 was expressed and purified as described before [66,67]. Briefly, single colonies WK6 *E. coli* with inserted NB237 sequence were inoculated in 15 mL Luria-Bertani Miller (LB) medium supplemented with 100 µg/mL ampicillin and incubated at 37 °C overnight with shaking at 200 rpm. Terrific broth medium supplemented with 100 µg/mL ampicillin, 0.1% glucose and 2 mM MgCl_2_ was inoculated with overnight cultures and incubated for 5 h at 37 °C with shaking at 180 rpm. Protein expression was induced by adding β-d-1-thiogalactopyranoside at final concentration of 1 mM. Bacterial cultures were incubated overnight at 28 °C with shaking at 180 rpm. Next day, bacterial cultures were centrifuged (8 min, 14 °C, 10,000 rpm), pellets were resuspended in Tris-EDTA-Sucrose (TES) and incubated for 1 h at 4 °C with shaking at 200 rpm. Osmotic shock was carried out by adding TES:dH_2_O (1:3) and pellets were incubated for 2 h at 4 °C with shaking at 200 rpm. After adding MgCl_2_ with a final concentration of 100 mM, the bacterial suspension was centrifuged (10,000 rpm) for 30min at 4 °C. Obtained periplasmic extract was mixed with Ni^+^-NTA agarose beads (Qiagen, 1 mL agarose beads per 1 L bacterial culture) and was incubated overnight at 4°C, shaking at 200 rpm. The next day, expressed nanobodies were purified with immobilized metal affinity chromatography, and eluted with 0.5 M imidazole in PBS. NB237 was then purified with size exclusion chromatography with ÄKTA pure system. The nanobodies were run on Sephadex 16/60 Hi-load column (GE Healthcare) using filtered and degassed PBS (pH = 9.10) as the mobile phase, with a flowrate of 1 mL/min. Protein concentration was estimated using the Unicorn software (GE Healthcare) on the ÄKTA purifier.

### 4.7. Invasion Assay In Vitro

GB cell lines U373 and U87 and NCH421k (GSC) invasion was measured according to the protocol described in Novak et al. [39], using 24-well Transwell units with 6.5 mm inserts and 8 µm pores (Corning, New York, NY, USA). U373 and U87 cells (10,000/insert) and NCH421k (80,000/insert) were seeded in the upper compartment, which was coated with 0.5 mg/mL Matrigel (Becton Dickinson, Franklin Lakes, NJ, USA) in serum-free medium. The lower compartment was filled with DMEM media containing 10% FBS. TRIM28 nanobody in a final concentration of 100 µg/mL was added into the upper chamber to GBs and to GSCs. The final time point for cell invasion was 48 h at 37 °C in 5% CO_2_. Invading cells were stained with 0.1% crystal violet and counted using the Nikon Eclipse Ti-inverted microscope (Nikon Instruments, Melville, NY, USA) at 4× magnification.

### 4.8. Zebrafish Embryo Model 

Wild-type AB zebrafish (*Danio rerio*) embryos were collected and incubated at 26 °C in dilution water (ISO 7346-3:1996). At 36 h of age, 0.005% phenylthiourea was added to the water to inhibit pigment formation. Zebrafish embryo xenotransplantation experiments were performed as described previously [34,35,36,59]. Briefly, xenotransplantation of 50 to 100 fluorescent GB cells (U87dsRED, U373eGFP) and GSCs (NCH421keGFP) in absence or presence of the TRIM28 nanobody (0.45 µg/mL) was performed by injecting 5 nL of cell suspension into the brain of embryos at 52h after fertilization with the MICROINJECTOR system (Tritech Research, USA). Embryos with implanted cells were incubated at 31 °C. For quantification of GB xenograft invasion and growth, fluorescence images of GB and GSCs in the embryos in lateral orientation were obtained at 1 day and 3 days after cell implantation, using an inverted fluorescence microscope (Eclipse T300; Nikon, Japan). The largest diameter and the diameter perpendicular to the largest of each GB xenograft were measured in images using ImageJ software (Software for image analysis, http://rsbweb.nih.gov/ij/; National Institutes of Health, Bethesda, MD, USA, accessed on 22 August 2021), and these two diameters were then averaged to obtain average GB xenograft length. GB xenograft length at 72 h was normalized to GB xenograft length at 24 h after xenotransplantation to determine the relative cell invasion. Tumour length is used to measure cell invasion because it is sensitive to cell dispersion, which means cell movement or invasion. While an increase in tumour length can also result from proliferation, we combined these results with results on GB xenograft fluorescence intensity to determine whether this is the case.

To determine differences in tumour growth, relative changes in mean fluorescence intensity of GB cells between 1 day and 3 days after cell implantation were quantified using ImageJ. The fluorescence intensity of xenografts was quantified in ImageJ by selecting the region of interest corresponding to the glioblastoma tumour on the basis of a fixed pixel intensity threshold value, and measuring the integrated density of the region of interest. The relative increase in fluorescence intensity, as a measure of tumour growth, was determined by dividing the measured values by the fluorescence intensity of the tumour in the same embryo at 1 day after implantation [35,36].

Relative cell invasion and tumour growth was compared between GB and GSCs alone and between GB and GSCs injected with nanobody, using Mann–Whitney test. ImageJ software (Software for image analysis, http://rsbweb.nih.gov/ij/; National Institutes of Health, Bethesda, MD, USA, accessed on 22 August 2021) was used to measure tumour size. All experimental protocols were approved by a National Institute of Biology. According to the EU Directive 2010/63/EU on the protection of animals used for scientific purposes, the earliest life-stages of animals, embryos up to 5 days, which were also used in this research study, are not defined as protected and; therefore, do not fall into the regulatory frameworks dealing with animal experimentation. Zebrafish facility and all procedures on zebrafish embryos and larvae are in compliance with the ARRIVE guidelines, all relevant national, international and EU legislation.

## Data Availability

All the data, supporting reported results can be provided upon request from the corresponding author.

## Supplementary Materials

The following are available online, Figure S1: *TRIM28* gene expression in glioma tissues and GB tissue subtypes. *TRIM28* mRNA expression levels were determined in GB (*n* = 156), non-cancerous brain tissues (N, *n* = 4) and in GB subtypes CL (*n* = 59), PN (*n* = 46) and MES (*n* = 51). Expression *TRIM28* values were analysed and visualized in GlioVIS database. (http://gliovis.bioinfo.cnio.es/, accessed on 22 August 2021). Figure S2: Kaplan–Meier survival curves for a cohort of 155 GB patients with high (red) and low (blue) expression of TRIM28. Figure S3. Kaplan–Meier survival curves for the two quartile cut-off high/low *TRIM28* expression in the top/bottom 25% of the samples. Figure S4: In vivo imaging of tumour growth in the brain of zebrafish embryos. Table S1: List of TaqMan^®^ assays used for RT-qPCR analysis (Thermo Fisher Scientific, USA). Table S2: Characterization of GB sample subtypes based on expression of the 15 genes.

## Sample Availability

Samples are available from the authors.
