# Supplementary material for: TRIM28 Selective Nanobody Reduces Glioblastoma Stem Cell Invasion
Source: Molecules. 2021 Aug 25;26(17):5141. doi: 10.3390/molecules26175141 (PMC8434287; doi:10.3390/molecules26175141)
Supplement: Supplementary file 1 [file molecules-26-05141-s001.zip › molecules-1309959-supplementary.pdf]

molecules

MDPI

Supplementary Materials

TRIM28 selective nanobody reduces glioblastoma stem cell invasion

Andrej Porčnik [1], Metka Novak [2], Barbara Breznik [2], Bernarda Majc [2,3], Barbara Hrastar [2], Neja Šamec [4], Alja Zottel [4], Ivana Jovčevska [4], Miloš Vittori [5], Ana Rotter [2], Radovan Komel [4,*], Tamara Lah Turnšek [2,6,*]

1 Department of Neurosurgery, University Medical Centre Ljubljana, 1000 Ljubljana, Slovenia; andrej.porcnik@ukclj.si (A.P.)
2 Department of Genetic Toxicology and Cancer Biology, National Institute of Biology, 1000 Ljubljana, Slovenia; metka.novak@nib.si (M.N.); barbara.hrastar@gmail.com (B.H.); barbara.breznik@nib.si (B.B.); bernarda.majc@nib.si (B.M.); ana.rotter@nib.si (A.R.); tamara.lah@nib.si (T.L.T.)
3 Jožef Stefan International Postgraduate School, 1000 Ljubljana, Slovenia
4 Medical Centre for Molecular Biology, Institute for Biochemistry and Molecular Genetics, Faculty of Medicine, University of Ljubljana, 1000 Ljubljana, Slovenia; ivana.jovcevska@mf.uni-lj.si (I.J.); neja.samec@mf.uni-lj.si (N.Š.); alja.zottel@mf.uni-lj.si (A.Z.); radovan.komel@mf.uni-lj.si (R.K.)
5 Department of Biology, Biotechnical Faculty, University of Ljubljana, 1000 Ljubljana, Slovenia; milos.vittori@bf.uni-lj.si (M.V.)
6 Faculty of Chemistry and Chemical Technology, University of Ljubljana, 1000 Ljubljana Slovenia
* Correspondence: T.L.T.: tamara.lah@nib.si; R.K.: radovan.komel@mf.uni-lj.si

TABLES

Table S1: List of TaqMan® assays used for RT-qPCR analysis (Thermo Fisher Scientific, USA).

Probe name	Gene name	assay ID	assay type	Cat.number
**GAPDH**	*GAPDH*	Hs99999905_m1	FAM-MGB, S(250rxns)	4331182
**HPRT1**	*HPRT1*	Hs02800695_m1	FAM-MGB, XS(75rxns)	4453320
**TRIM28**	*TRIM28*	Hs00232212_m1	FAM-MGB, XS(75rxns)	4448892
**COL1A2**	*COL1A2*	Hs01028956_m1	FAM-MGB, XS(75rxns)	4331182
**COL1A**	*COL1A*	Hs00164004_m1	FAM-MGB, XS(75rxns)	4331182
**TGFBI**	*TGFBI*	Hs00998133_m1	FAM-MGB, XS(75rxns)	4331182
**THBS1**	*THBS1*	Hs00962908_m1	FAM-MGB, XS(75rxns)	4331182
**DAB2**	*DAB2*	Hs01120074_m1	FAM-MGB, XS(75rxns)	4331182
**S100A4**	*S100A4*	Hs00243202_m1	FAM-MGB, XS(75rxns)	4331182
**P2RX7**	*P2RX7*	Hs00175721_m1	FAM-MGB, XS(75rxns)	4331182
**STMN4**	*STMN4*	Hs00229288_m1	FAM-MGB, XS(75rxns)	4331182
**SOX10**	*SOX10*	Hs00366918_m1	FAM-MGB, XS(75rxns)	4331182
**HER3**	*ERBB3*	Hs00176538_m1	FAM-MGB, XS(75rxns)	4331182
**ACSBG1**	*ACSBG1*	Hs00209500_m1	FAM-MGB, XS(75rxns)	4331182
**KCNF1**	*KCNF1*	Hs00266908_s1	FAM-MGB, XS(75rxns)	4331182
**ID1**	*ID1*	Hs03676575_s1	FAM-MGB, XS(75rxns)	4453320
**SOX2**	*SOX2*	Hs01053049_s1	FAM-MGB, XS(75rxns)	4453320
**CD44**	*CD44*	Hs00174139_m1	FAM-MGB, S(250rxns)	4331182
**OLIG2**	*OLIG2*	Hs00377820_m1	FAM-MGB, XS(75rxns)	4331182

Table S2: Characterisation of GB sample subtypes based on expression of the 15 genes.

molecules

Sample	Subtype	*COL1A2*	*COL1A*	*TGFB1*	*THBS1*	*DAB2*	*S100A4*	*P2RX7*	*STMN4*	*SOX10*	*ERBB3*	*ACSBG1*	*KCNF1*	*OLIG2*	*NOTCH*	*NF-KB*
s001	CL	0,010743	0,037266	2,11509	0,039042	0,760453	0,550467	0,910709	0,54942	0,253518	0,164841	1,642265	7,522426	1,840483	1,398655	1,044742
s002	CL	0,022497	0,081461	1,224671	0,063549	1,422315	0,928091	0,937	0,806889	0,200276	0,304841	2,436495	6,499148	2,079257	1,87655	0,804075
s003	CL	0,030444	0,06344	1,227904	0,386213	0,578327	1,047543	0,481217	0,305306	0,011655	0,048439	3,86452	20,13352	1,745498	1,608218	0,973727
s004	CL	0,702752	1,466638	1,828178	0,170579	1,45851	1,511108	0,496075	0,144497	0,015017	0,054485	5,186715	2,285376	0,626281	2,490112	0,952573
s005	CL	0,016712	0,030106	1,196495	0,040828	0,28123	1,200177	0,240501	0,037146	0,001187	0,008603	2,968765	15,21424	0,372059	0,772149	0,633171
s006	CL	0,017704	0,028778	1,495544	0,024088	1,604888	1,900744	2,15123	2,446527	1,326397	0,263375	2,919774	1,545422	5,842775	3,960549	1,391409
s007	mix	0,876903	1,77348	1,451737	0,681141	1,316531	0,870173	0,38851	0,572874	0,100016	0,058973	0,456403	0,91292	NA	0,85655	1,937338
s008	mix	0,026965	0,106624	0,734912	0,090394	1,506625	0,886789	0,419285	0,108025	0,026852	0,028492	0,544383	2,298997	0,17622	0,455887	1,050226
s009	PN	0,013569	0,025849	0,92241	0,236047	1,249694	1,262799	1,515741	4,167097	0,059174	0,131645	1,62002	1,267204	40,86526	5,56947	1,702867
s010	CL	0,00773	0,059795	1,722949	0,126674	0,945503	0,664747	1,463461	0,948559	0,246336	0,085554	2,192844	7,011375	1,430553	3,337731	2,684161
s011	CL	0,002046	0,011593	0,992169	0,015331	1,028752	0,696569	0,530203	0,641838	0,030463	0,14358	2,45679	6,957769	2,784954	2,206164	1,554648
s012	mix	0,034775	0,01097	0,502031	0,022702	0,214502	0,374288	0,763597	3,222049	0,550935	0,818809	0,642457	0,569106	1,864008	2,171268	0,818807
s013	mix	0,005647	NA	0,496543	0,005452	0,13519	0,206847	1,5007	1,206942	1,328509	10,28074	0,934038	0,083609	0,345065	0,690321	1,466832
s014	CL	0,120781	0,297379	1,252142	0,525453	2,936847	0,784445	0,985969	NA	0,01017	0,430233	1,722773	2,821166	0,407732	0,74736	1,732473
s015	mix	0,144614	0,121722	0,653508	0,116634	0,488642	1,006193	0,111875	0,009543	NA	0,016811	1,156279	0,40288	0,252031	0,557699	0,834504
s016	PN	0,010622	0,004869	3,696373	0,009845	1,688523	0,752452	2,549226	14,41829	6,100389	1,453963	4,859812	3,311124	5,994735	3,075015	2,317953
s017	CL	0,035457	0,153516	1,854953	0,083594	2,798036	2,981741	0,792381	0,235894	0,152677	0,131058	2,474198	3,187105	1,381073	2,367617	2,13069
s018	CL	0,062338	0,345172	1,285005	0,16959	1,568448	0,83846	0,706737	1,474376	0,86961	0,229355	3,621238	3,964212	0,415488	1,006912	1,569175
s019	CL	0,124325	0,064741	2,093345	0,058946	1,948841	2,531989	1,190443	1,156693	0,00436	0,914814	1,001199	3,404616	5,883936	1,784224	1,976168
s020	PN	0,004922	0,003056	2,842288	0,004971	3,994608	0,088696	2,242342	4,99102	0,887788	1,646237	3,558184	2,023709	17,34632	4,262166	2,908399
s021	mix	0,019865	0,007569	0,886218	0,05875	0,238985	0,724528	0,903331	0,688064	1,187497	0,856964	1,268091	2,512177	3,29844	1,642758	0,854799
s022	PN	0,062692	0,005546	0,564392	0,087251	0,451744	0,452948	2,559271	3,005958	29,78835	16,23366	1,21094	0,56174	3,021844	1,654655	1,041388
s023	mix	0,044734	0,002706	0,738794	0,035533	0,159227	1,026214	0,832442	3,023443	1,94376	2,370506	0,920665	2,031218	2,373847	1,42014	1,023346
s024	CL	0,148014	0,144261	0,98368	0,134876	0,2081	1,108735	1,922213	0,963711	0,248148	5,380735	2,909518	8,918588	2,123012	3,469878	1,980662
s025	CL	0,368601	0,021031	0,664664	0,075973	0,238224	2,012019	0,45911	0,186721	0,055642	0,47459	1,262785	1,955335	0,389367	1,65283	3,540112
s026	mix	0,146119	0,006702	0,506281	0,330752	2,471195	0,76193	0,094952	NA	0,000241	0,000001	0,046727	0,033886	0,000106	0,050205	0,40693
s027	MES	3,548609	4,020323	5,152646	4,803758	2,514586	0,328564	0,173316	0,001488	0,013709	0,037275	0,161527	0,551201	0,007262	0,329616	0,780085
s028	MES	2,808029	NA	1,843538	3,911333	4,544921	1,309628	0,126806	0,001799	0,060601	0,09824	0,067794	NA	0,000223	0,145182	0,855701
s029	mix	3,284406	1,737785	1,89136	3,419077	1,230599	0,429004	0,390276	NA	0,014047	0,047377	0,428341	0,02842	0,000553	0,432634	1,02534
s030	MES	0,875305	0,787815	1,233981	10,17887	1,906662	1,118611	0,944818	0,024995	NA	0,145513	0,988446	0,18414	0,009033	0,397877	1,840235
s031	mix	0,000053	0,000288	0,128633	0,000106	0,004705	0,037703	0,557328	0,016812	3,567281	0,887209	0,300095	0,271744	3,148641	0,952609	1,218073
s032	mix	NA	0,002257	0,134876	0,000505	0,028799	0,023457	0,915631	0,11737	3,784293	0,312452	0,012144	0,395012	0,827448	0,630509	0,332666
s033	mix	0,000857	0,000701	0,154858	0,002003	0,160556	0,087685	0,526643	0,002081	2,445774	0,409992	0,185162	0,181809	1,167068	0,378827	0,760749
s034	mix	0,000841	0,001048	2,075362	0,015142	0,130668	0,025353	0,116845	0,005321	0,000016	0,001919	0,053126	1,947658	1,582536	0,229594	0,59781
s035	CL	0,069213	0,086003	2,962944	0,058371	0,61556	0,457094	0,282799	0,823246	0,103053	0,041878	0,095625	1,697724	1,266418	1,401271	5,189586
s036	mix	0,103284	0,000164	0,405701	0,001475	0,07905	0,014256	0,669506	0,143895	0,060016	0,360519	0,39504	1,21738	0,825365	1,324922	0,741346
s037	mix	0,017463	0,001018	0,301104	0,010998	0,096898	0,625959	1,006514	3,498168	3,899864	4,359523	2,18127	1,724127	1,648217	1,58282	0,72716
s038	CL	0,218869	0,024723	1,516912	0,374283	0,823548	2,0851	0,8225	0,566622	2,095191	0,832297	1,533387	0,645374	0,27235	1,318332	1,35806
s039	CL	0,129195	0,363283	2,40977	0,159954	3,862967	1,264544	0,725185	3,820558	1,286329	0,345714	2,26798	0,23963	0,35801	0,812023	1,724255
s040	CL	0,1361	0,153832	1,618258	0,108231	1,589296	2,166598	1,657394	NA	2,364336	1,437268	0,750889	0,313309	2,766898	2,388643	0,951594
s041	MES	10,20754	8,061938	1,577088	0,709202	4,932083	0,935034	0,541706	0,010387	0,001263	NA	0,005287	2,81084	NA	0,447096	0,904167
s042	mix	0,79876	2,247731	2,359017	1,828887	0,541063	0,764274	0,438518	0,029358	0,00888	0,112456	1,180543	2,786372	0,845132	1,084014	0,953197
s043	mix	0,491172	0,342263	0,789067	0,199107	0,534845	0,417754	0,634038	1,013619	0,3305	0,251565	0,713472	1,199158	3,609955	0,723067	0,689825
s044	mix	0,096446	0,170519	0,35207	0,120508	0,14781	0,083381	0,462004	0,551022	1,540362	1,051473	0,834152	1,021205	0,705231	0,384685	0,301943
s045	PN	0,177215	0,386861	0,450285	0,222484	0,45718	0,129717	0,883595	1,50923	4,345073	3,923473	0,500091	0,949333	7,825623	6,291376	0,666993
s046	mix	1,440269	1,753406	0,729538	0,267022	0,153227	0,264099	0,391298	0,351019	0,026414	0,701012	0,950706	1,161139	1,832965	1,677818	0,793034
s047	mix	0,022628	0,041685	0,75995	0,151866	0,395881	0,33706	0,69415	0,288272	2,27368	0,500617	0,106729	0,151358	1,465795	1,233821	1,702291
s048	mix	0,505827	0,398706	1,160799	0,038305	0,448506	0,427517	0,685112	0,184248	NA	0,168631	3,069818	0,618881	2,502464	1,247276	1,162164
s049	mix	0,087467	0,575067	0,272	0,083428	0,081168	0,207171	0,233369	0,051701	0,005995	0,106855	0,295024	1,145973	1,318919	2,409882	0,679848
s050	mix	0,612476	0,58487	1,143947	2,336123	1,550948	0,399927	0,862297	0,320209	0,571766	0,271783	0,078308	0,08372	0,381178	0,212951	1,313305
s051	CL	3,169283	1,151673	1,048953	3,978698	1,217193	1,419639	0,560678	0,320613	0,027658	0,082101	0,37633	0,181767	0,170577	0,799669	3,090328
s052	PN	0,111348	0,12249	0,272517	0,129591	0,125537	0,107342	2,005383	0,87984	9,389925	3,700291	0,22763	0,460295	8,984523	1,797205	0,68204
s053	MES	16,2825	22,73361	1,738287	4,221793	1,547018	0,724009	0,953069	0,251027	0,233099	0,261286	0,880892	0,544448	1,172895	1,905386	1,159295
s054	MES	9,936406	6,619534	0,815623	1,32067	0,450517	0,897199	0,428553	0,098484	0,059288	NA	0,711829	0,027896	0,230391	0,318308	0,650022
s055	mix	0,217452	0,120248	0,211068	0,139229	0,176735	0,134491	0,089068	0,002335	0,007996	0,006137	0,070231	0,114201	0,021953	0,143418	0,760049

s056	PN	0,862298	1,192813	1,029128	1,139829	1,168131	0,339635	2,739298	NA	7,993863	8,490283	0,566048	1,26396	3,58491	1,172535	1,033517
s057	mix	0,181043	0,190897	0,686966	3,169078	0,524575	0,401592	1,160981	0,363036	2,057589	1,041521	0,128055	0,056239	3,77515	0,88432	0,710062
s058	mix	0,262477	0,696914	1,101054	0,387038	1,310428	0,558471	0,576851	0,266811	0,593552	0,524727	0,746754	2,166797	1,575117	1,135341	0,855542
s059	mix	0,082398	0,237925	0,664262	0,046585	0,299383	0,366682	0,33153	0,066544	0,000009	0,123422	1,255611	3,047229	1,625821	1,864591	0,889711
s060	mix	1,074574	2,631891	0,80808	2,734228	0,719507	0,460708	1,237693	0,420469	0,015728	0,747095	0,765016	1,458319	2,084383	2,067902	1,748391
s061	mix	0,208731	NA	1,012583	1,062996	1,071655	0,535799	2,190692	1,151323	4,635234	4,120888	0,668173	0,183448	2,592221	1,110753	0,913997
s062	mix	0,385735	NA	0,426794	4,00968	0,602637	0,248551	0,316615	0,06627	0,054572	0,124204	0,62503	0,253169	0,283168	0,355341	0,570901
s063	mix	0,036391	NA	0,078435	0,172357	0,134401	0,045953	0,087233	0,274462	NA	0,002875	0,003726	0,004255	0,003453	0,028104	0,614451
s064	mix	0,084188	0,062439	0,354535	0,062908	0,159256	0,135486	0,526629	0,56925	0,930005	0,48103	0,611675	0,297804	1,323476	0,641402	0,506385
s065	mix	1,184316	1,866429	1,075497	0,57469	0,602535	0,285197	0,219173	0,010685	0,014271	0,057914	0,187867	0,209685	0,019596	0,229271	0,680522
s066	mix	0,050828	0,214499	0,733315	0,173904	1,190459	0,128759	1,755458	0,545233	1,05903	0,732707	0,808537	0,162175	2,592359	1,259427	0,573841
s067	mix	0,343874	1,066514	1,098354	1,850936	1,988219	0,572003	0,595568	0,055602	0,247926	0,176846	0,25176	0,075545	0,116637	0,281702	0,641783
s068	mix	0,230862	0,680995	0,841127	0,081599	0,512926	0,434964	1,652073	0,125941	0,019868	0,088435	0,750503	0,083695	0,418519	0,341346	0,96208
s069	mix	0,066743	NA	0,097214	0,179912	0,149957	0,033124	1,216294	0,312725	0,259274	1,054611	0,164098	0,03663	3,036047	0,622913	0,299773
s070	mix	0,071398	0,313173	0,192793	0,148757	0,186974	0,040477	0,339236	0,208187	0,122338	0,384324	1,012747	0,92193	0,776556	0,291994	0,565369
s071	mix	1,3306	NA	0,850872	3,088279	1,289536	0,688404	1,944081	0,425186	0,030598	3,25883	0,721703	1,215601	3,594798	1,68744	1,030808
s072	mix	0,069547	0,243343	0,603077	0,283719	0,390708	0,428797	0,685105	0,347434	0,343034	0,669447	0,590891	0,664133	0,774717	0,448713	0,519476
s073	mix	0,132169	0,363041	0,148462	0,421712	0,369017	0,09637	1,742337	0,844865	5,076047	6,273282	0,087082	0,115106	7,038962	1,081501	0,579174
s074	mix	0,042721	0,093967	0,152266	0,225073	0,120598	0,059234	0,575541	0,374252	2,748757	2,560817	0,102663	0,138427	2,089298	1,019025	0,44415
s075	mix	0,099321	0,127221	0,777468	0,046102	0,107486	0,164655	0,528506	0,241765	0,064808	0,128763	3,314569	4,283057	2,825923	0,829932	0,707406
s076	mix	1,073856	1,106569	1,044049	0,715408	0,394943	0,773833	0,620467	0,228315	0,304654	0,257542	0,973971	0,63288	0,476906	0,840908	0,776479
s077	CL	0,087388	0,041475	1,15721	0,006168	0,238219	0,153679	1,410668	0,840641	0,209983	0,244411	1,793384	3,123404	0,053103	4,361258	1,489326
s078	mix	0,162963	0,470515	1,008945	0,684158	1,436005	0,779455	1,356962	1,935989	2,986893	2,104729	1,00126	0,115111	0,853715	0,861146	0,865285
s079	mix	0,044768	0,096433	0,522892	0,084185	0,289527	0,108959	0,250401	0,318356	0,011743	0,088794	1,134881	2,487453	0,838022	1,040966	0,642422
s080	mix	0,004481	0,003628	0,221574	0,011542	0,058926	0,016069	0,845747	1,287107	2,194599	1,73867	0,61123	0,466298	0,751917	0,173531	0,252088
s081	mix	0,265682	0,984022	2,445476	3,479037	0,904549	0,713911	NA	0,029389	0,015461	0,022929	0,384755	1,728724	0,137022	0,299233	0,640945
s082	mix	0,248708	0,149975	0,401489	0,032201	0,124214	0,051441	0,375918	1,107428	0,78606	0,76698	1,463318	0,596435	0,327214	0,624401	0,235083
s153	mix	1,813703	0,233101	1,28324	0,336045	0,553727	0,185377	0,988789	0,772857	3,669929	0,820831	1,146612	0,235705	2,469413	1,514754	0,703353
s154	mix	0,183829	0,045675	1,091757	0,077492	0,227911	0,114859	1,254826	0,469508	1,118144	0,743747	1,157249	0,347208	0,984801	0,425086	0,390933
s156	CL	0,340722	0,876858	2,52071	0,151144	1,406724	2,943165	1,023388	0,459678	0,129754	0,464113	1,023491	2,355747	1,468188	3,178459	1,177528
s157	mix	0,115033	0,256299	0,959145	0,787653	0,415018	0,219543	0,54452	0,599925	0,285346	0,936032	0,362552	0,678212	1,529926	0,999926	0,457483
s158	mix	0,719599	0,742103	0,911627	1,891873	0,753908	0,390317	0,435278	0,58244	0,256491	0,210921	0,204894	0,255919	0,335654	1,169141	0,823789
s159	mix	1,388617	0,518442	0,906261	0,090919	0,364521	0,221914	0,711978	0,598255	0,17056	0,685924	1,060647	1,559844	1,583484	2,146948	0,755996
s160	mix	0,083955	0,423422	1,663894	0,738615	0,622259	0,31139	0,385045	0,081428	0,083464	0,412962	0,083164	0,282849	0,503991	0,317186	0,740798
s161	PN	0,083971	NA	2,342812	0,197455	1,282831	0,191804	4,560177	8,299317	10,95946	6,012898	1,516455	0,072288	3,82944	0,406282	0,754017
s162	mix	0,409477	0,807339	0,844271	0,918276	1,008117	0,206438	0,82124	0,494484	2,387228	0,708497	0,229847	0,127907	1,299369	0,429136	0,893916
s163	mix	0,079505	0,266316	0,653007	0,086517	0,191137	0,185198	0,527909	1,67599	0,444364	0,270779	0,239124	1,338345	1,340001	0,491821	0,266051
s164	PN	0,081427	0,385398	1,772612	0,0485	0,341012	0,065416	3,653293	4,933502	6,621232	5,972783	1,174062	0,153662	2,4633	0,325319	0,742077
s178	MES	14,19216	26,21212	1,563311	1,012146	1,002156	1,446352	0,357625	0,081374	0,049058	0,049993	0,025945	0,784206	0,00723	0,346451	0,678689

molecules

MDPI

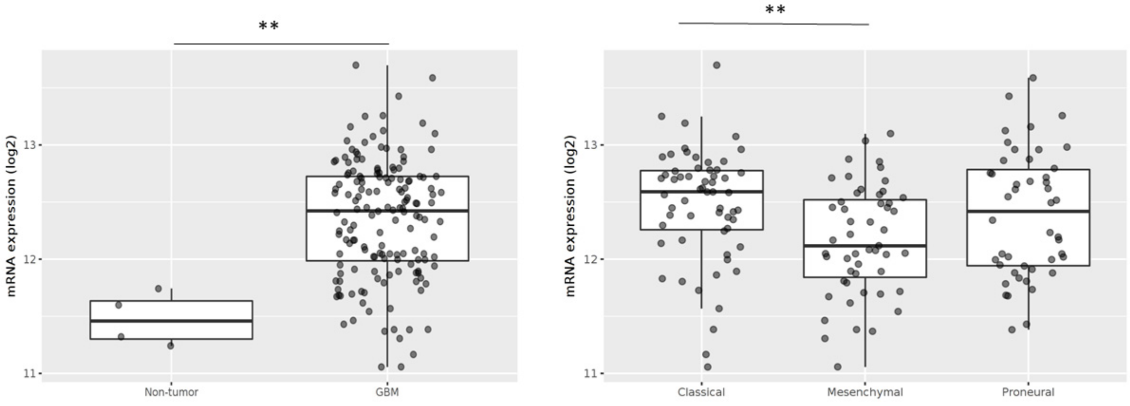

Figure S1. *TRIM28* gene expression in glioma tissues and GB tissue subtypes. *TRIM28* mRNA expression levels were determined in GB (n=156), non-cancerous brain tissues (N, n=4) and in GB subtypes CL (n=59), PN (n= 46) and MES (n=51). Expression *TRIM28* values were analysed and visualised in TCGA GlioVIS database. (http://gliovis.bioinfo.cnio.es/). Tukey's post hoc test was used for multiple comparison between groups with significance levels (** P < 0.01).

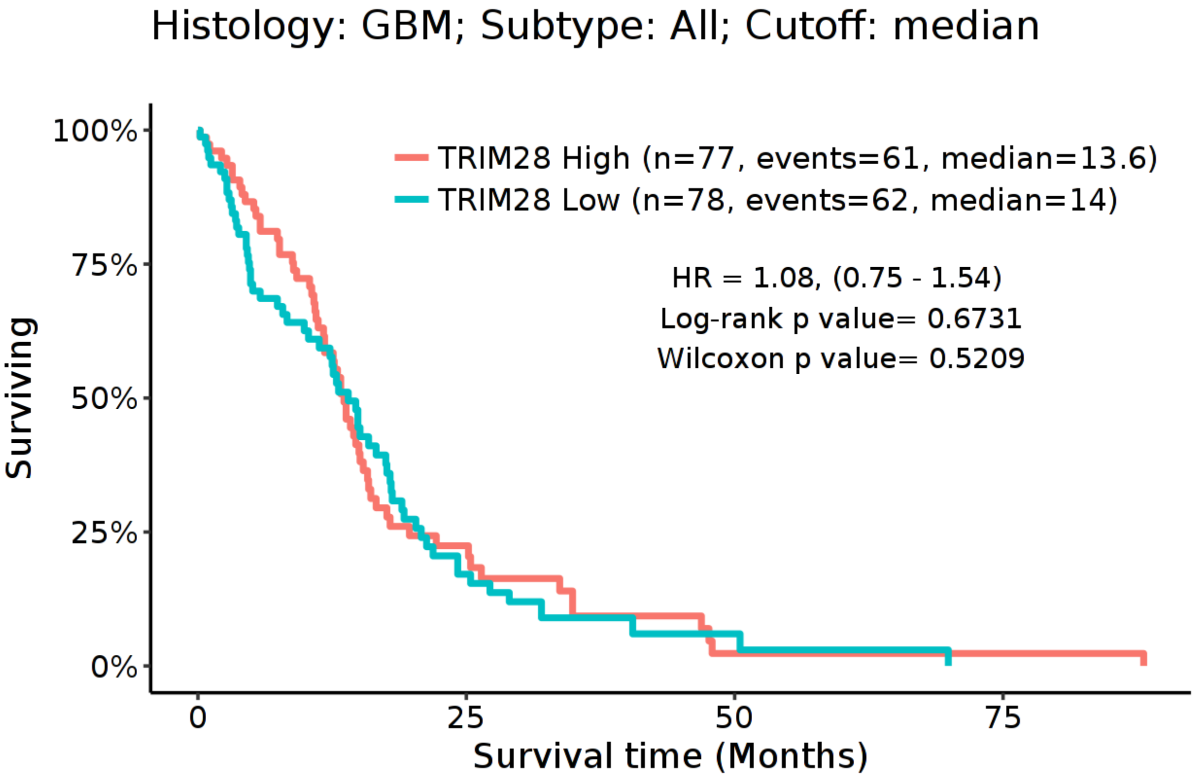

Figure S2. Kaplan–Meier survival curves for a cohort of 155 GB patients with high (red) and low (blue) expression of *TRIM28* based on median gene expression value analysed and visualised in GlioVIS database.

molecules

MDPI

(http://gliovis.bioinfo.cnio.es/). *TRIM28* is not related to survival rate in this validation cohort of patients, similar to our experimental cohort (Fig.3).

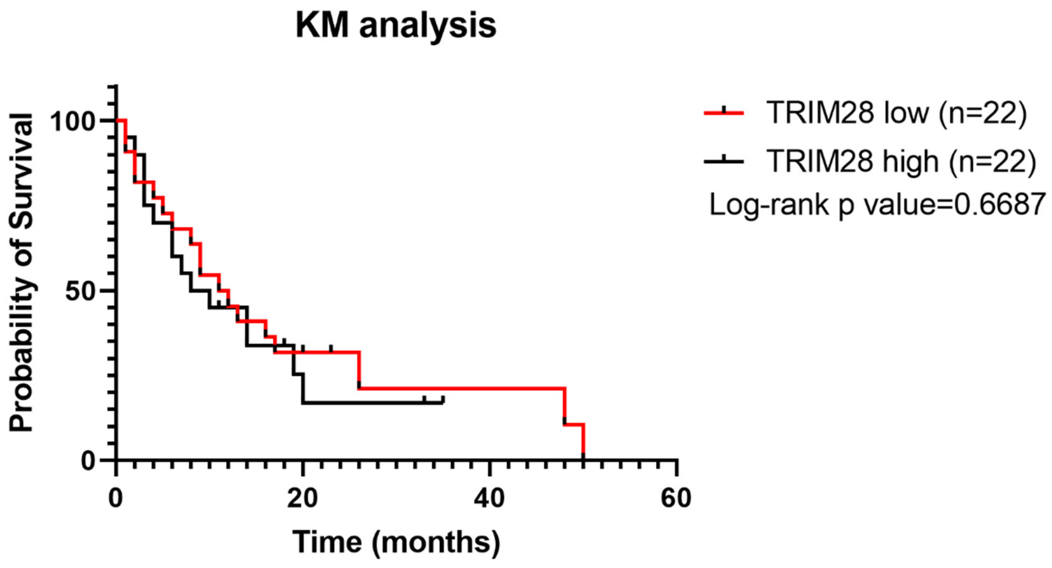

Figure S3. Kaplan–Meier survival curves for the two-quartile cut-off high/low *TRIM28* expression in the top/bottom 25% of the samples. Kaplan–Meier survival curves for a cohort of 44 GB samples with high (black line)/low (red line) *TRIM28* expression in the top/bottom 25% of the samples. *TRIM28* expression is not related to survival rate in our cohort of patients. Kaplan–Meier survival curves analyses were performed using GraphPad Prism software.

molecules

MDPI

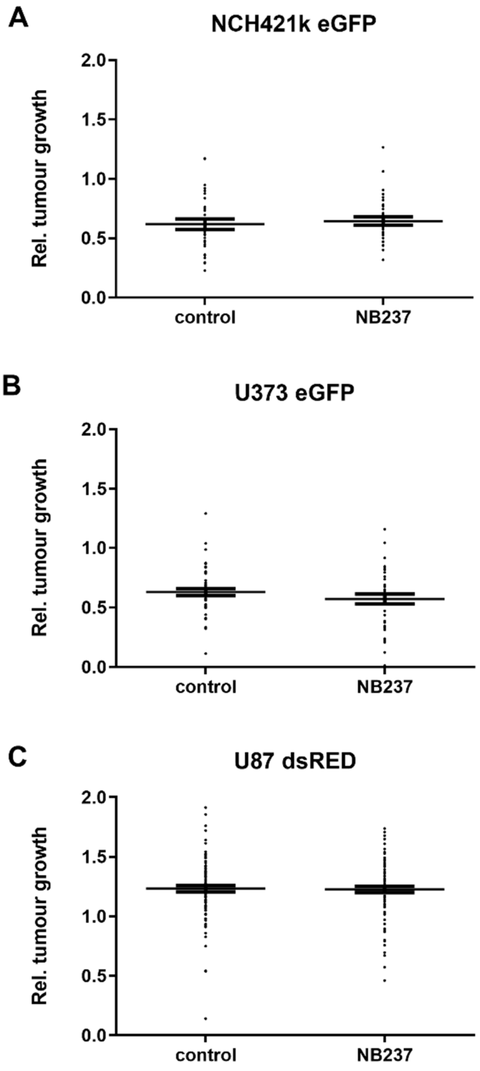

Figure S4. In vivo imaging of tumour growth in the brain of zebrafish embryos. Embryos 72h after the xenotransplantation of (A) NCH421k eGFP, (B) U373eGFP and (C) U87DsRed in the brain (visible as red and green fluorescence) without (control) or with the TRIM28 nanobody NB237. Data are shown as means ± S.D., dots on graphs A, B and C represent each embryo. Fluorescence intensity of GB xenograft at 72h was normalised to fluorescence intensity of GB xenograft at 24h after xenotransplantation to determine relative tumour growth. Number of embryos in control and with NB237 in the experiment with U373 n=37-50, U87 n=94-108, NCH421k n=13-38. Statistical analyses were performed using GraphPad Prism software, using Mann–Whitney test.
